# Putative avian teeth from the Late Cretaceous of Alberta, Canada, are more likely from crocodilians

**DOI:** 10.1371/journal.pone.0283581

**Published:** 2023-03-28

**Authors:** Sydney R. Mohr, John H. Acorn, Philip J Currie

**Affiliations:** 1 Department of Biological Sciences, University of Alberta, Edmonton, AB, Canada; 2 Department of Renewable Resources, University of Alberta, Edmonton, AB, Canada; Università di Roma, ITALY

## Abstract

Isolated teeth, previously referred to Aves, are more common than other bird fossils from the Late Cretaceous of Alberta. However, there are no known morphological synapomorphies that distinguish isolated bird teeth, and features of these teeth are generally shared with those of non-avian theropods and crocodilians. Here, specimens ranging from Late Santonian to Late Maastrichtian in age are described and qualitatively categorized into morphotypes, most of which strongly resemble teeth of extant juvenile and some fossil crocodilians. Variation within this sample of teeth may therefore reflect the heterodont dentition of crocodilians, rather than avian species diversity. Quantitative analysis Principal Component Analysis was mostly uninformative, with limited overlap between putative avian teeth and those of known Cretaceous birds, crocodilians, and non-avian theropods. The reassignment of these putative avian teeth to Crocodylia has important ramifications for our understanding of the evolutionary history of Cretaceous birds.

## Introduction

Birds are sparsely represented in Late Cretaceous fossil assemblages from Alberta. They appear as isolated postcranial elements, especially coracoids [1–5], as well as isolated teeth [6]. Most non-dental avian material from Alberta has been referred to *Hesperornis*, *Ichthyornis*, and *Palintropus* [1–3], and provisional ornithurine taxa, although most remain Ornithurae indet. [3–5, 7]. Only a few of the isolated teeth bear close resemblance to *Ichthyornis* or *Hesperornis*. The latter are recurved, with fine longitudinal ridges extending from the base of the crown to the apex.

The practice of referring isolated teeth to Aves began with Sankey et al. [6], who described teeth with straight, triangular, labiolingually compressed, and basally indented crowns,with or without carinae on their mesial and distal margins, and lacking denticles. On some, denticles or crenulations (that superficially resembles denticles) are present [8, 9]. Sankey et al. [6] identified teeth as avian based on similarity to *Hesperornis*. However, these teeth also resemble those of related non-avian theropods such as *Microraptor zhaoianus*, and the tooth-based genus *Richaroestesia*. The posterior teeth of *Microraptor* are constricted between the root and crown, as in troodontids and toothed birds [10–12]. Similar teeth from the early Maastrichtian of Alberta have since been identified in a vertebrate microfossil assemblage dominated by terrestrial taxa [13]. Currie and Coy [8] describe a single tooth with crown and root as avian, perhaps hesperornithid, although this identification has since been reconsidered [14, 15]. Longrich [7] describes possible bird teeth from the Maastrichtian Lance Formation of Wyoming. However, the Wyoming teeth seem too large for any known contemporaneous bird taxon.

Larson and Currie [15] and Larson [16] summarize the challenges associated with identifying small isolated theropod teeth, including the presence of similar characters in distantly-related taxa, and spatial and temporal gaps between specimens assigned to the same taxon. Tooth identification is also complicated by heterodonty and ontogeny. Despite recent advances in the evaluation of isolated theropod teeth [17–19], these challenges persist when identifying isolated teeth.

Nonetheless, numerous features have been proposed as diagnostic or synapomorphic for bird teeth. Currie [10] suggests that a basal constriction between the root and crown might be a synapomorphy for the teeth of birds and troodontids, but Dumont et al. [14] noted that various other theropods also possess this feature. An expanded root, also proposed as an avian feature, is also present in other maniraptorans [12]. Currie and Koppelhus [20] identify bird teeth by their bulbous crowns, but it is now apparent that *Hesperornis*, *Ichthyornis*, and most putative avian teeth from the TMP and UALVP collections lack this feature [14]. While dental synapomorphies for the dentition as a whole were identified for Avialae [12], avian dental synapomorphies are unknown for isolated teeth.

Assessing the taxonomic identity of the putative bird teeth from Alberta has important ramifications for avian diversity, evolution, and extinction in the Late Cretaceous. For this reason, this study attempts to test whether these “cf. Aves” teeth are more similar to *Hesperornis* and *Ichthyornis*, non-avian theropods, or juvenile crocodilians, both fossil and extant.

## Institutional abbreviations

TMP, Royal Tyrrell Museum of Palaeontology, Drumheller, Alberta, Canada; UALVP, University of Alberta Laboratory for Vertebrate Palaeontology, Edmonton, Alberta, Canada; UAM, Alabama Museum of Natural History, University of Alabama, Tuscaloosa, Alabama, USA; YPM, Yale Peabody Museum, New Haven, Connecticut, USA.

## Materials and methods

Sixty-four isolated teeth, initially catalogued as avian, were examined in the TMP and UALVP collections. Teeth were recovered through screen-washing or surface collection from numerous sites and formations across Alberta [6]. These include the Milk River (Santonian-Campanian), Oldman (Campanian), Dinosaur Park (Campanian), St. Mary River (Campanian-Maastrichtian), Horseshoe Canyon (Campanian-Maastrichtian), and Scollard (Maastrichtian) formations (Table 1). Localities included Devil’s Coulee, Dinosaur Provincial Park, Dry Island Buffalo Jump Provincial Park, Iddelsleigh, Milk River Valley, Onefour, and Tolman, Alberta, Canada. In most cases, more exact stratigraphic data are not available.

**Table 1:** Stratigraphic distribution of morphotypes.

Formation	Morphotype
Scollard	1, 7, 10
Horseshoe Canyon	1, 6, 9
St. Mary River	3
Dinosaur Park	1, 2, 3, 4, 5, 6, 8, 9, 11
Oldman	4, 5, 6, 7, 8, 10
Milk River	2, 8, 9

Stratigraphic distribution of morphotypes, spanning the Late Santonian/Early Campanian to latest Maastrichtian of the Late Cretaceous in Alberta.

List of measured isolated teeth from the Royal Tyrrell Museum (TMP) and University of Alberta Laboratory of Vertebrate Paleontology (UALVP) collections placed in their respective morphotypes, based on crown shape, enamel ornamentation, and presence or absence of denticles.

**Fig 1:**
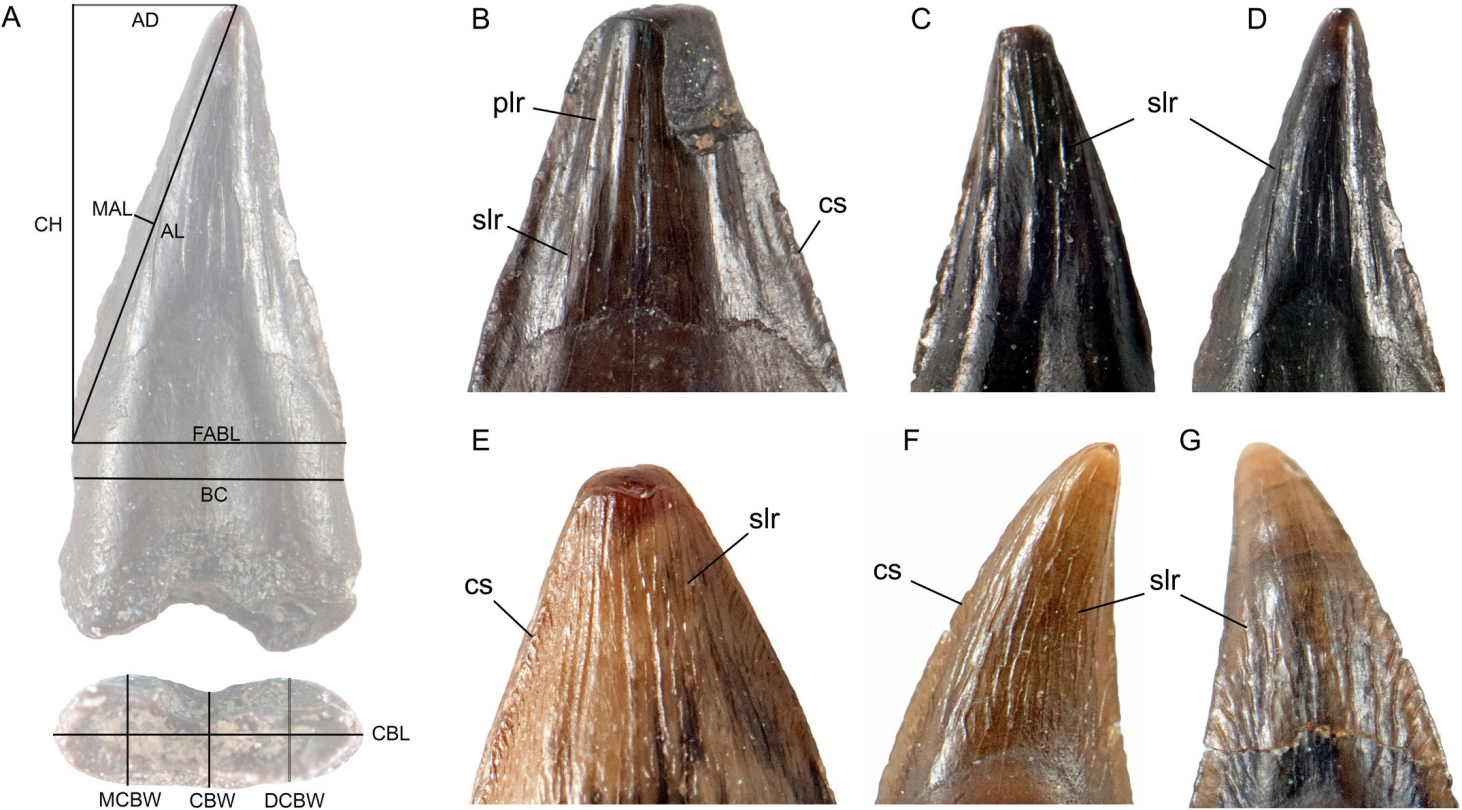
Tooth measurements and details of surface features on the crowns of putative avian and crocodilian teeth. **A**, isolated “bird” tooth (TMP 1995.184.20C) with tooth measurements superimposed. Lateral view: **AD** (apical distance), **CH** (crown height), **MAL** (mesial-apical distance), **AL** (apical length), **FABL** (fore-aft basal length), **BC** (basal constriction). Basal view: **CBL** (crown basal length), **CBW** (crown basal width), **MCBW** (mesial crown basal width), **DCBW** (distal crown basal width). **B**, TMP 1999.24.152 (morphotype 1); **C**, UALVP 852–4 (Morphotype 2); **D**, TMP 1995.184.20C (Morphotype 2); **E**, UALVP 54359A (fossil crocodilian); **F**, **G**, lingual and labial views of uncatalogued C (Morphotype 3). Abbreviations: cs, carinal striae; plr, primary longitudinal ridges; slr, secondary longitudinal ridges. Images not to scale.

**Fig 2:**
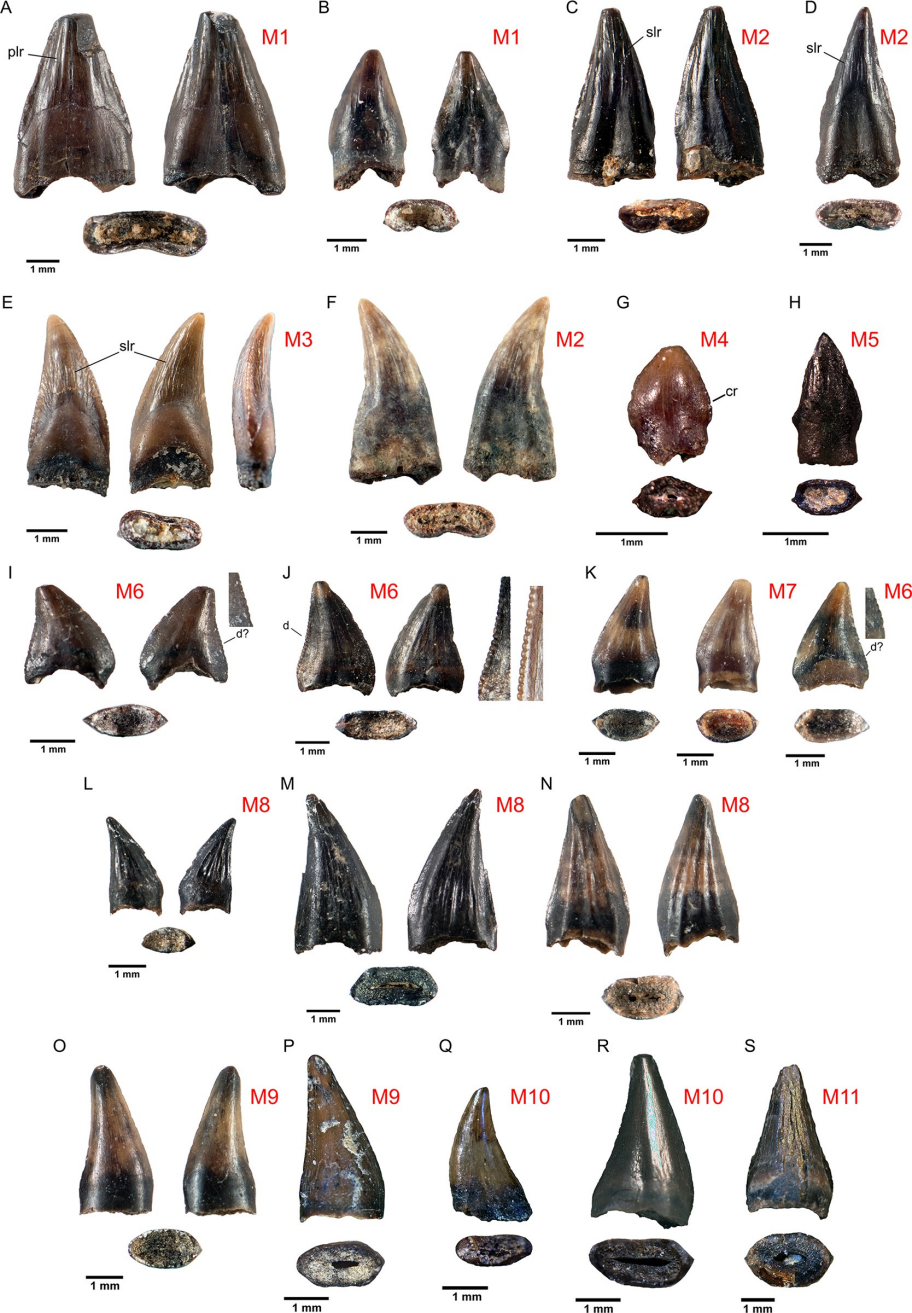
Putative isolated avian tooth morphotypes A, B, Morphotype 1; C, D, F, Morphotype 2; E, Morphotype 3; G, Morphotype 4; H, Morphoytpe 5; I, J, Morphotype 6; K, Morphotype 7; L, M, N, Morphotype 8; O, P, Morphotype 9; Q, R; Morphotype 10; S, Morphotype 11. Abbreviations: cr, crenulations, d, denticles (d? indicates possible denticles), plr, primary longitudinal ridges, slr, secondary longitudinal ridges. For A, B, C, E, F, I, J, K, L, M, N, and O, left is labial, right is lingual. E includes mesial aspect. D, G, and P are in lingual view, and H, Q, R, and S are in labial view. Specimen numbers are listed in S1 File.

Specimens were examined, measurements taken (Fig 1), and arranged into distinctive morphotypes based on characteristics of the crown, including overall shape in lateral and basal views, curvature, features of the enamel surface, and presence or absence of denticles on the mesial and/or distal carinae (Fig 2 and S1 File). Exemplars for each morphotype were selected and photographed. Terms for tooth shape and ornamentation were modified after [21, 22].

Partial or highly fragmented specimens were excluded from the analysis, and a total of 64 putative avian teeth were measured (S1 Appendix and S1 File). As well, we examined teeth of *Richardoestesia gilmorei* (TMP 1988.091.0028), *Troodon* sp. (TMP 1986.177.008), *Hesperornis regalis* (YPM 1206B), and *Ichthyornis dispar* (UAM_PV93.2.133_2). These were chosen for their relatively small size, general resemblance, and local provenience. We also examined fossil crocodilian teeth including UALVP 54359A, and four teeth from TMP 1986.008.0074. Modern material comprised five juvenile *Alligator mississippiensis* teeth (UAM2 R949, 6.3 cm skull length) and four teeth of *Caiman crocodilus* (uncatalogued juvenile specimen from the University of Alberta Zoology Museum; 16.25 cm skull length). Specimens were examined using a Nikon SMZ1500 stereomicroscope, and photographed in lateral and basal views using a Q-imaging Retiga 4000r Fast 1394 digital camera at 50x magnification. Images were processed and measurements were performed in NISElements BR 3.0. Additional photographs were made with a Nikon D500 camera, a Nikkor AF-S 150mm micro lens set at f22, a Nikkor TC-17E II teleconverter, and a Nikon 5T two-element close-up lens. Additionally, three teeth of a juvenile *Crocodylus niloticus* were measured from published images [14] using ImageJ [23].

Most tooth measurements (Fig 1A) were based on previous studies [6, 15, 21, 22], as follows. Fore-aft basal length (FABL) was measured as the maximum mesiodistal length of the base of the crown. Crown height (CH) was measured perpendicular to FABL, from the mesial edge of FABL to the apex of the crown. As the tips of most teeth were worn, CH was measured to the existing worn apex, and not to an extrapolated point in space. Apical length (AL) was measured diagonally from the mesial end of FABL to the approximated midpoint of the worn apex.

Novel measurements were also assessed. Apical distance (AD) was measured perpendicular to CH from the crown apex to the CH line, as a measure of the displacement of the apex relative to the crown base, such that values of AD roughly equal to 0.5 X FABL represent straighter crowns. Greater values of AD represent strongly rearward-directed (recurved) crowns. Mesial-apical length (MAL) provides a measure of the convexity of the mesial carina, measured as the maximum distance from the AL line to the mesial carina. Basal constriction (BC) of the tooth below the crown was measured as a minimum horizontal distance below and parallel to FABL. In basal view, two additional measurements were modified from [21, 22]: Crown basal length (CBL) is the maximum mesiodistal length of the tooth or root. In many instances this value is identical to FABL, but on some teeth the length of the crown and root differ (Fig 1A). Crown basal width (CBW) was measured across the center of the tooth, perpendicular to CBL. To better capture variations of shape in basal view, mesial crown basal width (MCBW) and distal crown basal width (DCBW) measurements were made at points halfway between CBW and the ends of CBL. In most cases, one of these measurements was the maximum width value for the tooth as a whole. In the few instances in which it was not, the maximum width value differed by only 0.01–0.04 mm from either MCBW or DCBW. Measurements of CH, CBL, and CBW for *Hesperornis* and *Ichthyornis* were incorporated into our analysis.

Collectively, these ten measurements characterize the basic geometry of a tooth. Because principle coordinate analysis (PCA) has been applied to similar problems [3, 15–19, 21, 24], we chose to employ this technique as well. PCA ordination requires continuous variables, however, so discrete variables such as enamel ornamentation were not included in the analysis. Values were normalized prior to performing PCA in PAST version 3.15 [25].

## Results and discussion

### Morphotype descriptions

The sample was arranged into 11 more-or-less distinctive morphotypes, and these were characterized qualitatively as follows (Fig 2 and Table 2).

**Table 2:** List of specimen numbers with their corresponding morphotypes.

Morphotype	Catalogue #	# Specimens per Morphotype
**1**	TMP 81.31.96, TMP 95.184.20A, TMP 97.96.26, TMP 99.24.152, TMP 2009.22.92, TMP 2009.137.19, TMP 2009.163.81A, TMP 2014.6.244B, UALVP 57560D	9
**2**	TMP 94.184.20B, TMP 95.184.20C, TMP 2009.25.12, UALVP 852-4, UALVP 57560A, UALVP 57560B	6
**3**	TMP 95.168.13B, TMP 2014.6.244A, UALVP 57560C	3
**4**	TMP 96.6250A	1
**5**	TMP 94.144.114, TMP 96.62.54A	2
**6**	TMP 86.9.96, TMP 87.158.76, TMP 95.174.52, TMP 95.181.66A, TMP 95.181.66E, TMP 95.181.66G, TMP 2000.6.2, TMP 2009.22.29B	8
**7**	TMP 86.6.2, TMP 95.143.57, TMP 95.151.21, TMP 95.168.13A, TMP 96.177.79B, TMP 95.181.60A, TMP 95.181.60H, TMP 96.66.52A, TMP 96.62.53A, TMP 96.62.53B, TMP 96.62.54B, TMP 96.62.56A, TMP 96.62.63, TMP 2000.53.60, TMP 2001.36.6, TMP 2009.22.91	16
**8**	TMP 95.177.79, TMP 95.181.66D, TMP 95.181.66F, TMP 95.181.66J	4
**9**	TMP 86.21.68, TMP 86.33.56, TMP 86.45.27, TMP 87.4.46, TMP 95.181.66C, TMP 95.181.66I, TMP 96.62.51, TMP 96.62.55B, TMP 96.62.55C, TMP 96.62.62, TMP 2000.45.52, TMP 2003.57.2, UALVP 483	13
**10**	TMP 96.62.52B, TMP 2003.89.33, TMP 2009.163.81B	3
**11**	TMP 95.181.66B, TMP 96.1.14	2

#### Morphotype 1

Morphotype 1 is characterized by large size (maximum height 4.56 mm), a tall profile, an elongate shallow lingual groove, and two types of ridges on the enamel surface (Fig 2A and 2B). Primary longitudinal ridges are relatively straight, broad, rounded ridges roughly equal in size and extending from the base of the crown to the apex. Secondary longitudinal ridges are fine, short, and typically wavy, running roughly parallel to each other and concentrated on the middle and apex of the crown. These longitudinal ridges differ from the grooves on the teeth of the early Cretaceous enantiornithine birds of the Jehol formation in China. *Sulcavis* [26] and *Monoenantiornis* [27] both possess grooves on the lingual surfaces only, not raised ridges such as we see in the Alberta material. In general, no Jehol enantiornithines preserve striations, ridges, or denticles on their teeth [26].

The mesial and distal carinae are also subtly textured, with very fine and closely-spaced wavy striae that extend apically from the crown onto the carinae. These differ from the larger, straighter, and wrinkle-like marginal undulations described by [22], which also do not appear to occur on the carinae. The carinae are prominent and continuous along both mesial and distal margins. The base of each carina slopes onto the crown, giving the crown a pinched appearance. The carinae terminate abruptly at the base of the crown, below which the root may be very slightly constricted. The crown varies from straight to slightly recurved, and the mesial and distal margins are more or less straight in lateral view. The lingual surface possesses a deep, wide groove that narrows towards the apex. In better-preserved specimens, the root can be wider than the crown. The enamel surface, both lingual and labial, has an arched appearance, below which the surface is largely featureless. Basally, the tooth is reniform in cross-section and labiolingually compressed. This type was featured in Sankey et al. [6] (see Fig 5 [35–38]), and similar teeth were described from the Maastrichtian Lance Formation by Longrich [7] (see Fig 9A, 9B, 9D in Longich [7]).

#### Morphotype 2

These teeth closely resemble morphotype 1, but the central groove on the lingual surface is restricted to the base of the crown, rather than extending towards the apex. Teeth possess prominent, textured mesial and distal carinae, and a series of primary longitudinal ridges, secondary longitudinal ridges, or both (Fig 2C, 2D and 2F). The crown is also slightly recurved, and is narrower than Morphotype 1. The carinae are continuous from crown base to apex. A slight indentation is present ventral to the base of the carinae, and the roots are typically expanded. In cross-sectional view, the teeth are compressed and reniform. This type was also described by Longrich [7] (see Fig 9C, 9E in Longrich [7]), Sankey et al. [6] (see Fig 5 [39–42] in Sankey et al. [6]), and Gates et al. [28].

#### Morphotype 3

Morphotype 3 is similar to Morphotype 2, but smaller and with a marked lingual curvature (Fig 2E). Morphotype 3 is represented by three teeth: TMP 2014.6.244A, TMP 1995.168.13B, and UALVP 57560C. Densely spaced and often irregular secondary longitudinal ridges are apparent on both TMP 1995.168.13B and UALVP 57560C, but less so on TMP 2014.6.244A (some features may have worn away). The ridges on the labial surface are larger, more irregular, and splay toward the carinae, whereas those on the lingual surface are straighter, longer, and extend towards the apex. Primary longitudinal ridges are absent. A small constriction is present beneath the base of the carinae, and the expansion of the root is visible in most examples. Basally, TMP 2014.6.244A and UALVP 57560C are reniform, and widest mesially. TMP 1995.168.13B is similar but less reniform.

#### Morphotype 4

Morphotype 4 is represented by a single tooth (TMP 1996. 62.50A); the smallest in the sample (1.2 mm in height) (Fig 2G). The crown is low and rounded, with convex mesial and distal margins in lateral view and a strong basal constriction. Like Morphotypes 1, 2, and 3, it has fine, crenulate, apically-directed ridges on the carinae. Primary and secondary longitudinal ridges are absent. The base of the crown is relatively conical and lacks a broad groove. In basal view the tooth is widest mesially, and lenticular.

#### Morphotype 5

Morphotype 5 is represented by two teeth: TMP 1994.144.114 (Fig 2H) and TMP 1996.62.54A. The very small crown (2 mm in height) is relatively straight, pointed, and basally somewhat swollen, with prominent carinae and subtle primary longitudinal ridges. The basal constriction is prominent. In basal cross-section the tooth is compressed labiolingually and subrectangular to oval in shape, and widest mesially. TMP 1996.62.54A is slightly more basally elongate than TMP 1994.144.114.

#### Morphotype 6

Morphotype 6 has a low profile (Fig 2I and 2J), and the mesial margin is strongly curved basally, contributing to a constriction between crown and root. In some specimens, the distal margin slopes outwards basally to form a small, angular shoulder. The crown is recurved and carinae are either thin and apical, or absent. Denticles are present on the margins, although these are typically not continuous from base to apex. A shallow groove on the labial and/or lingual base of the crown is apparent on some specimens. Some specimens, such as TMP 1987.158.76, have a slightly reniform cross-section, most are lenticular to parlinon (with linguoanteriorly- and linguoposteriorly-angled margins) in shape, and widest anteriorly. Sankey et al. [6] (see Fig 5 [43–46]) describe a tooth similar to TMP 1995.181.66G, with denticles on the posterior margin.

#### Morphotype 7

Morphotype 7 resembles Morphotype 6 except for the absence of denticles (Fig 2K). The teeth are recurved with a strongly convex mesial margin. Like Morphotype 6, a small, angular shoulder is often present at the base of the distal margin, beneath which the crown and root are constricted. The base of the mesial margin is either sharply angled or smoothly curved. Some are slightly concave near the apex of the mesial and distal margins (e.g., TMP 1996.181.60H). Low carinae are typically present from the base to the apex on both mesial and distal margins. The enamel surfaces are smooth, lacking ornamentation on the labial and lingual faces. Preservation is variable, and the root is expanded in lateral view on some but not all specimens. Basal outlines also vary, although all are labiolingually compressed and elliptical to lenticular, trapezoidal or weakly figure-8 shaped (arachiform).

#### Morphotype 8

A distinctive tooth with prominent primary longitudinal ridges (Fig 2L–2N), although these are more poorly developed in TMP 1995.181.66D. Much like Morphotype 2, but not reniform in basal cross section. In lateral view, the crown is tall, narrow, and recurved with a rounded mesial margin and a relatively straight distal margin. Small carinae are present on both margins, somewhat lingually. Carinal striae are absent. The base of the crown is moderately rounded and gently slopes towards the weak basal constriction. TMP 1996.181.66J has a more pronounced basal shoulder on the mesial margin, projecting past the root of the tooth. Basal cross-sections vary from rectangular to elliptical, figure-8 shaped, or slightly lenticular.

#### Morphotype 9

In lateral view these teeth are tall and recurved, similar to Morphotype 8 but with smooth enamel surfaces (Fig 2O and 2P). The mesial and distal margins are basally rounded and straight to slightly curved. Carinae vary in strength, and are smooth and typically offset lingually, giving some cross-sections a parlinon shape. Others are oval or rectangular to figure-8 shaped. On other specimens, the carinae are situated on the midline. There are faint striations on the carinae of TMP 2000.45.52. Teeth are moderately labiolingually compressed and a basal constriction is weak to absent in lateral view.

#### Morphotype 10

Morphotype 10 is angled somewhat mesially but also recurved (Fig 2Q and 2R). TMP 2003.89.33, identified as unique and potentially avian by Brinkman et al. [29], is especially so. Minute carinae are present on the mesial and distal margins. Labial and lingual surfaces are smooth and lack ornamentation, although a shallow, narrow indentation may be present at the base of the crown. There is no basal constriction, but the roots are slightly expanded. In cross-section the teeth are compressed and weakly reniform to figure-8 shaped.

#### Morphotype 11

Morphotype 11 is the most nearly conical of the morphotypes (Fig 2S) and is represented by two specimens, both of which are smooth, although both examples are worn. The bases of the labial and lingual faces are somewhat bulbous in mesial and distal view, and the mesial and distal edges curve inwards at the cervix, producing a slight constriction Minute carinae are present on the mesial and distal margins of TMP 1995.181.66B, and absent on TMP 1996.1.14. The teeth are weakly compressed and elliptical in cross-section.

### Comparisons with juvenile crocodilian teeth

**Fig 3:**
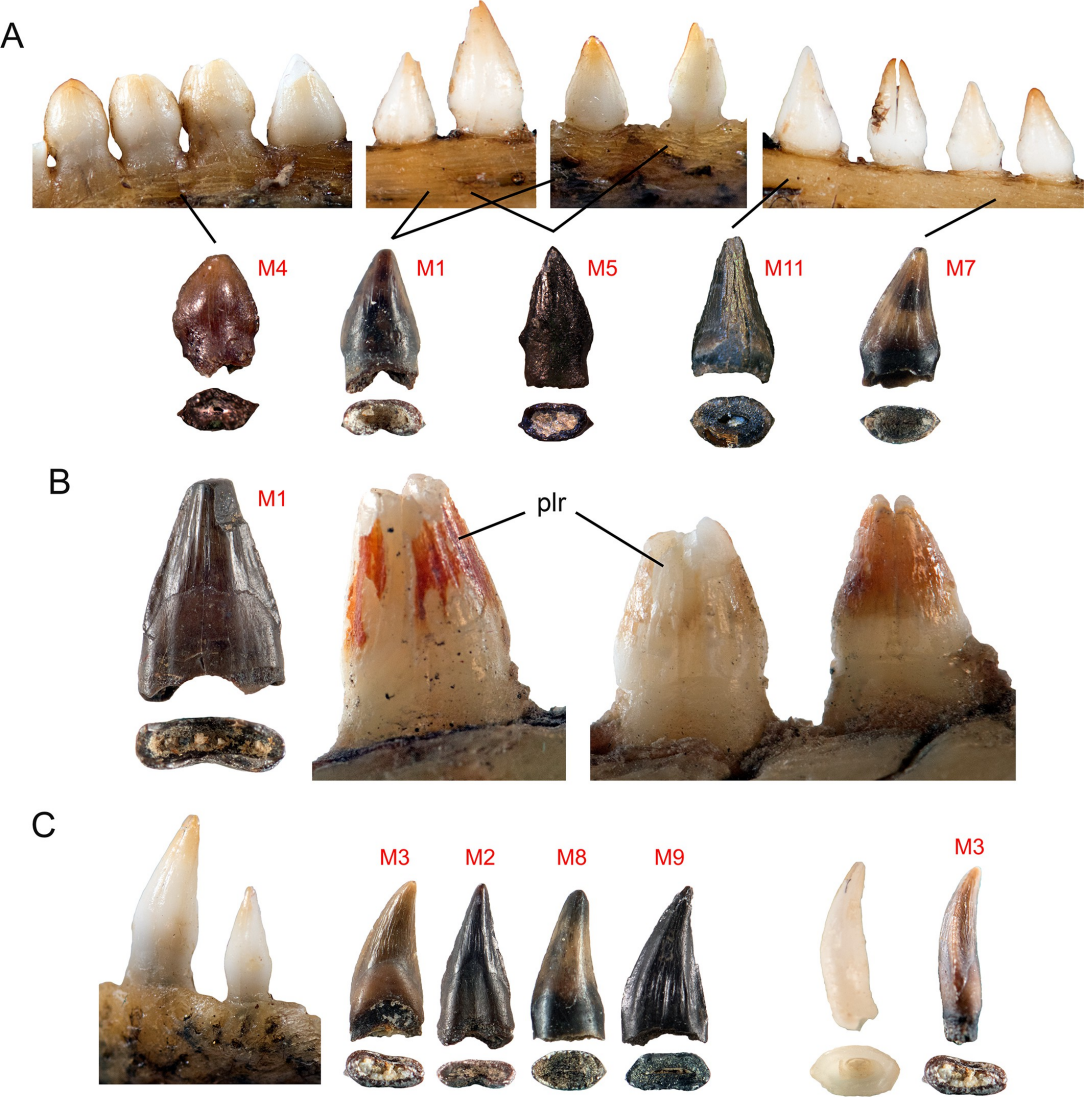
Comparison of putative fossil avian teeth with dentition of extant *Alligator* and *Caiman*. **A**, Left to right: posterior and mid-region maxillary teeth of juvenile *Alligator*, compared with Morphotypes 4, 1, 5, 11, 7; **B**, Morphotype 1 compared with *Caiman* teeth; **C**, Left, juvenile *Alligator* premaxillary caniniform teeth; Right, Morphotypes 3, 2, 8, 9, and comparison of *Alligator* caniniform tooth in mesial aspect with Morphotype 3. Abbreviation: plr, primary longitudinal ridges. Images not to scale.

**Fig 4:**
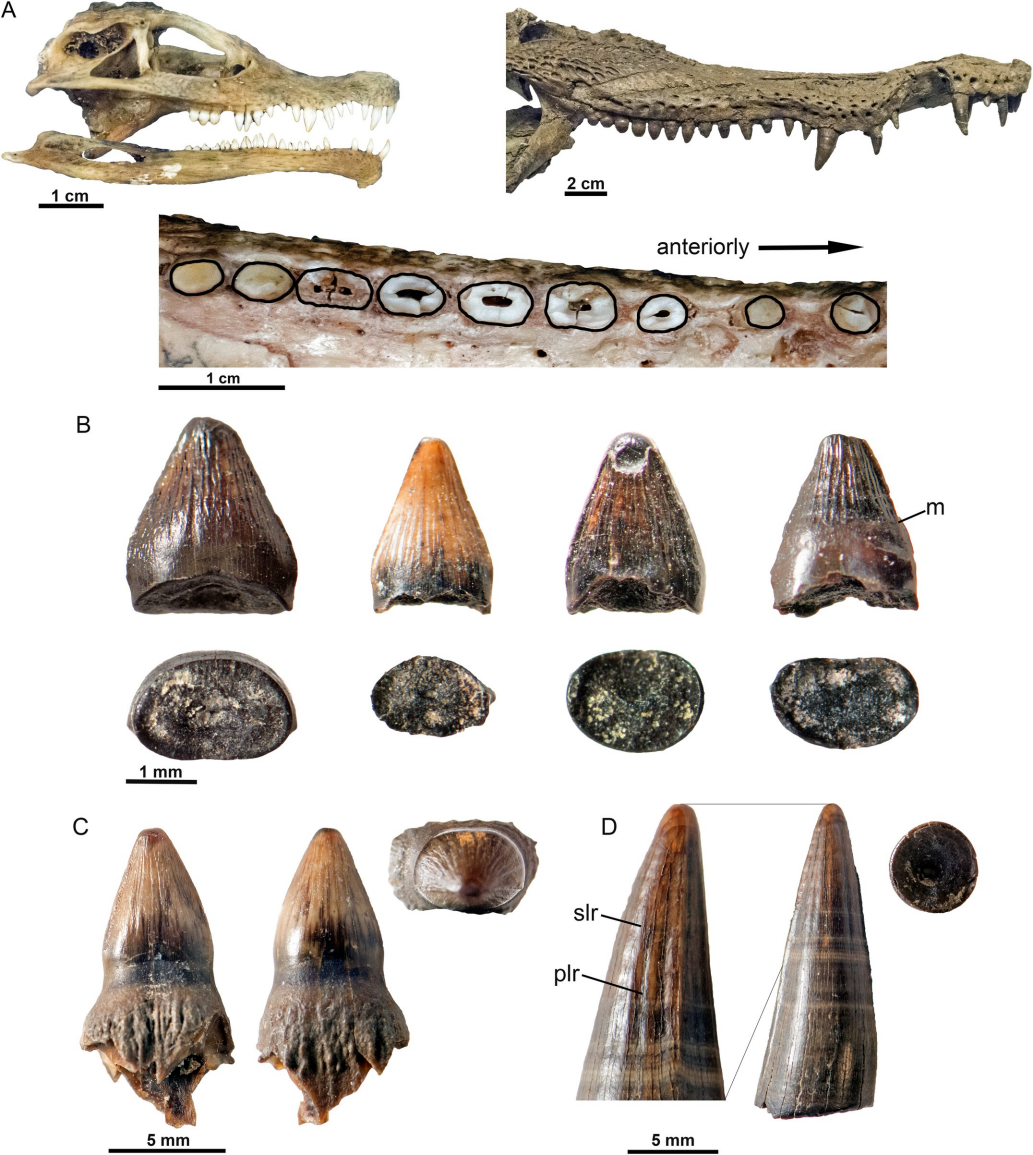
Variation of tooth shape in crocodilian jaws and small fossil crocodilian teeth from the Dinosaur Park Formation, Alberta. **A**, juvenile *Alligator* skull; **B**, skull of UALVP 40954 in lateral view; **C**, dorsal aspect of *Caiman* maxilla with tooth shape in apical view outlined in black; **D**, selection of teeth from TMP 1986.8.74 (presumably crocodilian) in lateral and basal views. Note the ridges present on all four crowns, and possible enamel margin; **E**, UALVP 54359A in lingual, labial, and apical views, **F**, UALVP 54359B in lateral and basal views, including details of ridges near the apex of the crown. Abbreviations: m, margin, plr, primary longitudinal ridges, slr, secondary longitudinal ridges. Crocodilian fossils, especially isolated teeth and scutes, are relatively common in Late Cretaceous assemblages from Alberta. However, none of the known fossils preserve jaws with teeth from young individuals. Very small crocodilian teeth, similar in size or smaller than those of the juvenile *Alligator* used in this study, are also unknown [29, 30]. Crocodilian taxa from Alberta include the neosuchian *Gilchristosuchus palatinus* from the Milk River Formation, the alligatoroids *Albertochampsa langstoni* and *Leidyosuchus canadensis* from the Dinosaur Park Formation, the alligatorine *Strangerochampsa mccabei* from the Horseshoe Canyon Formation, and the probable crocodyloid *Albertosuchus knudsenii* from the Scollard Formation [28, 31–35]. *Leidyosuchus* is the most abundant of these, and it is well-represented by skull and jaw material [33]. Unfortunately, the smallest specimen of *Leidyosuchus* (UALVP 53765) does not preserve teeth.

Many of the morphotypes closely resemble those of young crocodilians, and Morphotypes 1, 2, 3, 4, 5, and 11 are remarkably similar to various tooth positions in juvenile *Alligator* and *Caiman*, particularly in lateral aspect (Figs 3 and 4B). There are also similarities in mesial aspect with respect to Morphotype 3 (Fig 3C) and in basal cross-section for many of the morphotypes as well (Fig 4A and 4B). These comparisons are striking, and we believe that they provide strong evidence that the "c.f. Aves" teeth are in fact the undescribed teeth of juvenile crocodilians.

Crocodilian and known avian teeth possess similar enamel ornamentations, and both have fine ridges on the mesial and distal faces. Admittedly, putative bird teeth are more labiolingually compressed than is typical for adult crocodilians [7]. However, the teeth of juvenile *Alligator alligator* and *Caiman crocodylus* are labiolingually compressed and vary widely in cross-sectional shape (Figs 3A and 4A). The teeth of *Hesperornis* and *Ichthyornis* are typically conical, but range from lenticular to subcircular in cross-section, [14, 36]. Thus, degree of lateral compression does not allow us to distinguish avian from non-avian theropod, and crocodilian teeth.

Of the 11 morphotypes, nine closely resemble known crocodilian teeth, but the details of these similarities are complicated. In lateral view, Morphotype 1 resembles teeth midway along the maxilla and dentary of juvenile *Alligator* (Figs 2A and 2B and 3A and 3B); broad, with relatively straight mesial and distal edges, and with longitudinal ridges on both labial and lingual faces. Although Morphotype 2 is similar to some *Hesperornis* teeth (e.g., YPM.1206A, [14]), it is also similar to teeth in the premaxilla, anterior maxilla, and dentary of juvenile *Alligator*; narrow, tall slightly recurved, with a slight basal constriction (Fig 3C). Some examples are elliptical in cross section as are some small crocodilian teeth (Figs 2E and 4A). Carinae are present on Morphotypes 2 and 3, and on all crocodilians observed, (Figs 1B–1F, 3B and 4B). Primary and/or secondary ridges are present on Morphotypes 2 and 3 and in juvenile *Alligator*, *Caiman* and all fossil crocodilian teeth (Figs 1B–1G, 2C-2F and 3B). Isolated fossil teeth from the Maastrictian Lameta Formation of India and nearly identical to Morphotypes 2 and 3 were identified as crocodilian [37], although they are larger in size and elliptical in cross section. Enamel ornamentation is most prominent on small crocodilian teeth (Fig 4B) and can be reduced or more difficult to discern on larger examples, such as the known *Leidyosuchus* (Fig 4C and 4D).

Although fine, irregular ridges are present on the teeth of *Hesperornis* [14, 38], these are dissimilar to the secondary longitudinal ridges on Morphotypes 1, 2, and 3 and crocodilian teeth (Figs 1B–1G, 3B and 4B). Carinae in *Hesperornis* are either absent or smaller than those of Morphotypes 1, 2, and 3 [38], and lack carinal striae. Primary and secondary longitudinal ridges and striated carinae are present on fossil and modern crocodilian teeth, but have not been described for any Mesozoic bird, so this particular combination of characters could potentially represent distinguishing features of crocodilian teeth.

The low, broad crown, and crenulate carinae of Morphotype 4 bear strong resemblance to crocodilian teeth in the posterior parts of the upper and lower jaws of *Alligator*, *Caiman*, and *Leidyosuchus* (Figs 2G and 3A). Similarly shaped teeth from the Nile Crocodile (*Crocodylus niloticus*) have also been illustrated in [39], in Late Cretaceous Crocodylia indet. [37], and in Paleocene *Asiatosuchus* sp. [40], all of which have crenulations or serrations along the mesial and distal carinae.

In lateral aspect, Morphotype 5 is similar to *Ichthyornis* [14], although missing the angled root, and small teeth with straight to slightly curved edges are also present in the middle portion of the jaws of juvenile *Alligator* (Figs 2H and 3A) as well as some other juvenile crocodile teeth [14], although these are somewhat more narrow. In basal view, Morphoptype 5 is broadly lenticular to elliptical and closely resembles juvenile *Alligator* and *Caiman*.

Morphotypes 6, 7, 8, and 9 share features with crocodilians, non-avian theropods, and some birds (Fig 2I–2P). However, none have been attributed to crocodilians [6, 15]. The denticles of Morphotype 6 resemble those of the ziphosuchian crocodyliform, *Doratodon ibericus* from the Campanian of Spain. Company et al. [41] compare these teeth to *Richardoestesia isosceles*, and suggested that the latter may be a crocodyliform rather than a non-avian theropod.

Morphotype 7 is also difficult to interpret. The degree of curvature varies, such that some are nearly straight and similar to teeth in the middle to posterior areas of crocodilian jaws, as well as isolated crocodilian teeth (Fig 3A). The lenticular cross-sections are also reminiscent of crocodilians, and more trapezoidal or figure-8 shapes are also seen in non-avian theropods [12].

Morphotype 8 is similar to the long, narrow, recurved teeth on the premaxilla, anterior and middle maxillae and dentaries of crocodilians (Figs 2L–2N and 3C). The crowns also bear primary longitudinal ridges although these are typically irregular, or restricted to the base or apex, as in the crocodilian teeth TMP 1986.8.74, UALVP 54359B, *Caiman*, and *Leidyosuchus* (Figs 3 and 4B-4D). The rounded mesial and distal edges of TMP 1995.191.66F resemble those of some *Hesperornis* teeth [42], although a similar shape is also present in juvenile *Alligator* (Figs 3C and 4A). Some examples, particularly TMP 1995.181.66J, also resemble the possible non-avian theropod *Paronychodon lacustris*, which also has a strong distal curvature, primary longitudinal ridges, and a projection mesial base of the crown [6]. The lenticular to elliptical and figure-8 shaped cross-section typical of Morphotype 8 is also present in *Caiman* (Fig 4C), other crocodilian teeth [37], *Paronychodon* [6, 28], and “*Paronychodon*-type” teeth, potentially synonymous with *Richardoestesia sp*. [7], but see [16].

The enamel surfaces of teeth attributed to Morphotype 9 are smooth, although some (eg.,TMP 2000.45.52) have a prominent carina with slight, angled ridges (Fig 2O and 2P). The isolated crocodilian tooth UALVP 54359B also shares this feature but is more conical in cross-section (Fig 4D). Smooth carinae and primary or secondary longitudinal ridges are shared by some non-avian theropods (*Paronychodon*), birds (*Hesperornis*) and crocodilians [6, 37, 38]. However, striated carinae and primary or secondary longitudinal ridges seem to be unique to crocodilians (Fig 2E–2G). Apically-angled striae are also present on the labiolingually compressed, unserrated teeth of “false-ziphodont” crocodilians such as the Paleogene *Asiatosuchus*, and the Late Cretaceous *Trematochampsa* [37]. In some examples, Morphotype 9 is tall, narrow and reminiscent of Morphotypes 2, 3, and 8 (Figs 2C, 2D, 2E and 2N-2P). Larson et al. [13] referred TMP 2003.57.2 to Avialae indet., but it and other examples of Morphotype 9 resemble non-avian theropod teeth [6, 7, 43, 44], including dromaeosaurs and *Richardoestesia*. Examples with straighter crowns are similar to long, narrow crocodilian teeth positioned anteriorly in the jaw (Figs 3C, 4A and 4B). A basal constriction is typically present, as in juvenile *Alligator* and *Caiman*, and some examples of *Richardoestesia* [7]. Norell et al. [45] describe a neonate dromaeosaur with non-denticulate teeth, but these are more conical, rather than labiolingually compressed. *Paronychodon* teeth are also non-denticulate [44, 46], and the presence of denticles on *Richardoestesia* can vary [6, 7]. In cross-section Morphotype 9 is less compressed than Morphotypes 1 and 2, and similar to the broad lenticular, elliptical, and figure 8-shaped shapes seen in *Alligator*, *Caiman*, and *Leidyosuchus* (Figs 3B, 3C, 4C). Rectangular and figure-8 shaped cross-sections are shared with most small non-avian theropods including *Richardoestesia* [6, 14, 37, 46].

Of the morphotypes identified here, Morphotype 10 seems most likely to be avian (Fig 2Q and 2R). Two small jaw fragments from the Cenomanian Greenhorn Formation of Kansas, containing teeth comparable to Morphotype 10, were diagnosed as avian by Bell and Everhart [47] based on the recurved, unserrated teeth and prevalence of foramina on the jaw. Like TMP 1996.62.52B, TMP 2003.89.33, TMP 2009.163.81B (all Morphotype 10), the Kansas teeth lack enamel ornamentation, and the mesial and distal edges are strongly curved. The teeth possess the swept-forward shape of Morphotype 10. Although they differ considerably in age, these teeth are markedly similar.

Finally, Morphotype 11 is also comparable to some teeth in the middle portions of upper and lower crocodilian jaws (Figs 1S, 3C, 4A and 4B), although lacking ornamentation. The straight mesial and distal edges and rounded base are most similar to juvenile *Alligator*. This morphotype may be among the most difficult to interpret as they are uncommon, nondescript, and typically worn.

### Principal component analysis

**Fig 5:**
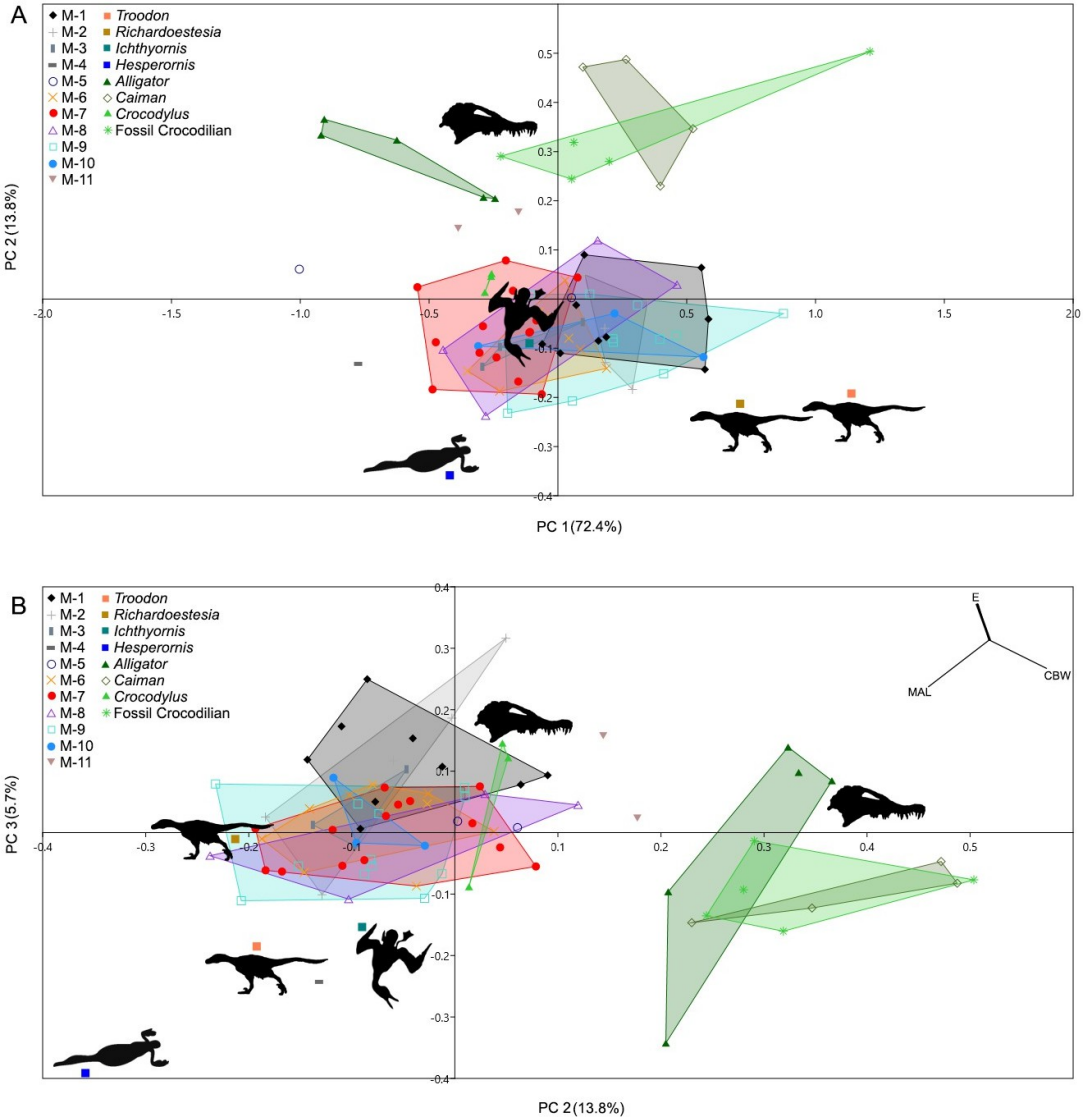
Principal Component Analysis of putative avian teeth morphotypes, selected non-avian theropod and bird teeth, and fossil and modern crocodilian teeth. **A**, Principal Component 1, x axis, Principal Component 2, y axis; **B**, Principal Component 2, x axis, Principal Component 3,y axis; Convex hulls are included around Morphotypes and taxa. Top right corner shows vectors for the following variables; CBW, crown basal widths; E, variables including AD, CH, BC, FABL; MAL, mesial apical distance.

PCA clustered most putative avian teeth together, separate from almost all other teeth of known identity, confirming the general sense that they are in some way different (Fig 5A and 5B)., The three specimens from Morphotypes 4 and 11, however, did not overlap with any of the other morphotypes. Both juvenile *Crocodylus* teeth and the single tooth of *Ichthyornis* grouped with the putative avian teeth (and with Morphotype 7 in particular) on the first two component axes, separate from juvenile *Alligator*, *Caiman*, fossil crocodilian teeth, *Hesperornis*, and *Richardeoestesia* (Fig 5A). Thus, with respect to the choice between avian and crocodilian affinities, this ordination is unhelpful.

It must be kept in mind, however, that not all of the features we examined could be quantified and included in the PCA, and not all continuous morphological variables could be measured on all teeth. As well, due to the approximately equal loading values of all variables for PC 1, this first component axis is interpreted as a composite index of size. The smallest teeth plot to the left and the largest plot to the right (Fig 5A). As is typical of morphological PCA, the second and third component axes are more informative with respect to differences in shape. On these axes, *Ichthyornis* and *Hesperornis* did not plot within the central cluster, while *Richardoestesia* and the crocodilians other than *Crocodylus* fell outside the central cluster. The overlap with juvenile *Crocodylus* teeth provides partial support for the crocodilian identity of the central cluster, while the separation of the remaining crocodilians on PC2 (but not PC3, except for one specimen) seems contradictory. However, crown basal width (CBW) is the main variable separating teeth on PC 3. In general, the wider, elliptical teeth of juvenile *Alligator*, *Caiman*, and fossil crocodilian teeth were not as strongly compressed as compared to isolated unknown examples from Alberta. Morphotype 11 (TMP 1995.181.66B and TMP 1996.1.14) possesses intermediate crown basal widths, and plots between the central and wide-based crocodilian clusters. With advancing ontogeny, we fully expect crocodilians to differ from the central cluster, and this may be evident in the PCA. A more ambitious ordination, involving the transformation of all relevant characteristics into continuous variables, and including teeth from various taxa worldwide, might resolve the taxonomic differences, but such an analysis is currently beyond the scope of our research.

## Summary and conclusions

Qualitatively, most of the teeth we examined strongly resemble those of juvenile crocodilians. Several morphotypes are closely comparable to particular tooth positions of both extant and fossil crocodilians, and variation among the morphotypes may represent variation within the dentition of particular taxa, rather than taxonomic diversity. Enamel ornamentation, in the form of longitudinal ridges and carinal striae, is shared between crocodilian dentition and several teeth from the Cretaceous of Alberta. Thus, with the possible exception of Morphotype 10, evidence for avian affinities is lacking.

Reassigning these specimens to the Crocodylia has consequences for our understanding of avian diversity in the late Cretaceous of Alberta and western North America. For example, isolated teeth, primarily from the Hell Creek Formation and similar to those from Alberta, were used in an analysis tracking disparity in tooth shape of non-avian theropods and birds over time [46]. Using tooth shape as a proxy for ecological niche diversity, the authors conclude that both non-avian theropods and birds were diverse and largely stable leading up to the mass-extinction event. We now suspect that this analysis may instead pertain to interspecific variation in crocodilian teeth and/or intraspecific variations within the jaws. From non-dental fossils, we know that there were numerous taxa of late Cretaceous birds in Alberta, but we are not in a position to identify these taxa from teeth, or for that matter, to assume that they even had teeth. It is possible that the paucity of Cretaceous bird teeth from Alberta is a consequence of the ornithurine avifauna having been largely edentulous.

Ideally, our qualitative and quantitative analyses would have aligned in support of the juvenile crocodilian hypothesis. As it stands the qualitative resemblances are striking, but the PCA is uninformative. It is possible, however, that alternative techniques might improve this alignment. For example, three-dimensional computed tomography scanning [48], photogrammetry [49], optical scanning microscopy [50], and synchrotron scanning [51] have been used to elucidate various aspects of dental morphology. Additionally, three-dimensional geometric morphometric analysis has the potential to clarify patterns of similarity not apparent with multivariate ordinations alone [52–54]. Geometric morphometrics have also been used in conjunction with machine learning algorithms [55], and thus the analysis of tooth shapes is a rapidly evolving field. Such techniques are beyond the scope of our study, and we are not entirely confident that they would resolve the question, but we invite other workers to apply them to the fossil tooth sample from Alberta. Of course, the simplest and most definitive answer could come in the form of one or more well-preserved skulls with in-situ teeth, and we look forward to the day when such fossils are eventually uncovered.

## Supporting information

S1 Appendix Supporting information. Includes explanations for excluded tooth measurements\, and comparisons ruling out the dentitions of other taxa including *Archaeopteryx*, non-avian Theropods, Ornithopods, and Choristoderes.
(DOC)

S1 File Raw measurement data of individual tooth specimens. (XLSX)
