# Supplementary material for: Putative avian teeth from the Late Cretaceous of Alberta, Canada, are more likely from crocodilians
Source: PLoS One. 2023 Mar 28;18(3):e0283581. doi: 10.1371/journal.pone.0283581 (PMC10047536; doi:10.1371/journal.pone.0283581)
Supplement: S1 Appendix — Includes explanations for excluded tooth measurements\, and comparisons ruling out the dentitions of other taxa including Archaeopteryx, non-avian Theropods, Ornithopods, and Choristoderes. (DOC) [file pone.0283581.s001.doc]

**Supplementary Information**

**Excluded measurements**

A number of measurements were initially made but not included in the analysis.

Studies of non-avian theropod teeth typically measure the density of denticles per unit of length (Smith et al., 2005; Larson, 2009; Larson and Currie, 2013), and/or the height and length of individual denticles (Sankey et al., 2002). However, in most cases, the denticles on putative bird teeth were irregular both in shape and extent on the carina, varying widely in size, length, and height. As a result, the number of denticles per given length was not readily discernable, and therefore it was not possible to consistently measure individual denticles with certainty for the purposes of analysis. Thus, the crowns of teeth with denticles were measured for shape in the same way as the other specimens.

Likewise, the presence or absence, and extent of the carinae on the anterior and posterior margins, were difficult to interpret and measure, due to wear and damage.

As well, an attempt was made to quantify the distance from the posterior concave curvature of each tooth, as the minimum distance from the posterior margin of the tooth to the midpoint of a line, extending from the posterior end of FABL to the apex the tooth. However, these measurements were found to vary only slightly, unlike anterior curvature, which was more informative. As a result, posterior curvature was excluded from the final analysis.

The distance from FABL to the basal constriction, or basal height (BH), was also excluded. In an exploratory PCA, this variable gave high loading values for the first two axes, but a review of the measurements suggested the high variance for this measurement resulted not from morphological disparity, but from lack of discrete landmarks for the exact placement of FABL and BC.

Basally, many teeth were somewhat curved along their long axis. For these teeth, an angle was estimated from the center point of the CBW and CBL lines through the anterior and posterior edges. This angle was omitted from the PCA as initial ordinations showed this variable contributed little to each component axis, and because excluding the angle did not affect the form of the ordination.

Ratios are often used in tooth analyses (Smith et al., 2005; Larson, 2009; Hendrickx et al., 2015), but because ratios include the raw measurements from which they are derived, and because these raw measurements were included in the analysis, ratios were excluded to ensure statistical independence and avoid potential mixing of data types and values.

**Qualitative comparisons of putative avian teeth with various taxa**

**The Dentition of *Archaeopteryx***

Teeth in *Archaeopteryx* are small and unserrated, with differences in shape of the crown and root between the premaxilla and maxilla, as well as between specimens (Howgate 1984; Mayr et al., 2007). Howgate (1984) describe the teeth of the Berlin and London specimens as similar in shape; they are peg-like and lack ornamentation of the crown, although in some instances small carinae may be present (Louchart and Pouech, 2017). The teeth of the Eichstätt specimen are also similar overall, although they are more consistently recurved along the jaw (Howgate, 1984). Additionally, Howgate (1984) states that the Berlin specimen lacks a basal constriction between the crown and root, although this feature was shown to be present to varying degrees in some teeth of the Berlin, Eichstätt, London, Münich, Solnholfen, and Thermopolis specimens (Louchart and Pouech, 2017). Howgate (1984) also noted a constriction roughly midway along the crown itself, and this was likewise reported in the Solnholfen and London specimens (Mayr et al., 2007). In spite of the variability among *Archaeopteryx* teeth, crown shape tends to be fairly low and broad at the base, with pronounced curvature of the crown occurring along the anterior edge and near the apex. This particular combination of shape in lateral view is not reflected in any of the morphotypes we identified in the Alberta sample of putative avian teeth.

**The Dentition of Non-Avian Theropods**

Currie and Coy (2008) report denticles on an putative avian tooth, although these denticles are less complex in shape compared to those of non-avian theropods. Structures referred to as crenulations occur along the posterior edge of the premaxillary teeth on a new specimen of *Longipteryx*, and are likewise less complex than thsoe of most theropods, and irregular in shape (Wang et al., 2015).

TMP 89.103.25 was originally diagnosed as a possible hesperornithiform, with denticles showing an enamel-covered dentine core (Currie and Coy, 2008). Examples of hesperornithiform teeth in Cumbaa et al. (2006), however, differ in having smoothly curved anterior and posterior edges on the base of the crown that slope more gently towards the basal constriction, rather than the sharply angled edges on TMP 1989.103.25. This specimen is now thought to instead belong to *Richardoestesia isosceles* following quantitative analysis and comparison with other theropod teeth (Larson and Currie, 2013; Dumont et al., 2016). Sankey et al. (2002) stated that *Richardoestesia* teeth can be difficult to tell apart from avian teeth, but can be distinguished based on a lack of a basal constriction in *Richardoestesia isosceles*, and a stronger curve and larger denticles in *Richardoestesia gilmorei*. Also, neither have the distinct hourglass cross-section of the base of the crown in avian teeth as described by Sankey et al. (2002). Longrich (2008), however, described a slight constriction at the base of teeth of *Richardoestesia cf. gilmorei* from the Lance Formation. Also, denticles in *Richardoestesia gilmorei* and *Richardoesterisa cf. gilmorei* are present on the posterior edge, although anteriorly denticles are either significantly smaller or absent altogether (Sankey et al., 2002; Longrich, 2008). This is similar to teeth in Morphotype 6, in which denticles are not visible on the anterior margin (Table 1, Fig. 1K). The serrations seen on the Alberta teeth are also simple in structure and irregular in shape, although it is difficult to discern whether or not a dentine core was present.

Dumont et al. (2016) suggested that avian-like teeth with denticles were likely non-avian theropods. Although some teeth are lower and broader than TMP 1989.103.25, the overall shapes of the crowns are similar, and these too may belong to non-avian theropods rather than birds. Dumont et al. (2016) also suggested teeth shaped similarly to TMP 1989.103.25 and *Richardoestesia* also likely belong to non-avian theropods. The teeth and jaws of very small or juvenile non-avian theropods from Alberta are unknown, and may represent at least some putative avian teeth.

**The Dentition of Ornithopods and Choristoderans**

Although a basal constriction and triangular crowns lacking in serrations are features that are shared between putative avian teeth and the premaxillary teeth of some ornithopods (Longrich, 2008), so-called avian teeth from Alberta are dissimilar in lacking a combination of a broad, rounded crown with a strong posterior curvature of the apex, and roots that are typically elliptical or circular in shape (Boyd, 2014). Ridges that encompass the whole of the tooth from the base to the rounded apex in *Thescelosaurus* are also absent in the Alberta sample.

Members of crocodilian-like Choristodera are represented by two genera for which tooth and jaw material has been described. *Champsosaurus* teeth from the Dinosaur Park and Horseshoe Canyon formations are typically tall and conical in shape with some lateral compression and either posterior or medial curvature. The enamel of the crown is striated and exhibits infolding near an expanded base (Gao and Brinkman, 2005; Matsumoto and Evans, 2016). Neither of of these features is evident in the sample of putative avian teeth. Although the basal-most portion of some putative avian teeth is not preserved, the lack of enamel infolding, presence of a basal constriction, frequent presence of a basal groove, and strong lateral compression eliminates *Champsosaurus* from consideration here. *Cteniogenys* teeth from the Oldman and Dinosaur Park formations can be more similar to the cf. Aves teeth than *Champsosaurus*, but the crowns are low, relatively straight and triangular with blunt, rounded tips, and a circular base with weak to no constriction. There are no grooves on the lingual and labial faces, which also tend to be finely striated but lacking enamel infolding (Gao and Fox, 1998; Gao and Brinkman, 2005). However, as juvenile dental material of Choristodera from Alberta has not been identified or described, it is unknown if the teeth of subadults were labiolingually compressed like those of crocodilians.

**References**

1. Boyd CA. The cranial anatomy of the neornithischian dinosaur *Thescelosaurus neglectus*. PeerJ, 2014; 2: e669.
2. Brinkman, DB, Braman, DR, Neuman, AG, Ralrick, PE, Sato, T. A vertebrate assemblage from the marine shales of the Lethbridge Coal Zone. In: Currie PJ, Koppelhus EB, editors. Dinosaur Provincial Park: A Spectacular Ancient Ecosystem Revealed. Indiana University Press, Bloomington, Indiana; 2005a. pp. 486-500.
3. Cumbaa, SL, Schröder-Adams, C, Day, RG, Phillips, A. Cenomanian bone bed faunas from the northeastern margin, Western Interior Seaway, Canada. In: Lucas SG, Sullivan RM, editors. Late Cretaceous vertebrates from the Western Interior. New Mexico Museum of Natural History and Science Bulletin, 2006; 35. pp. 139-155.
4. Currie, PJ, Coy, C. The first serrated bird tooth. In: Sankey JT and Baszio S, editors. Vertebrate microfossil assemblages, their role in paleoecology and paleobiogeography. Indiana University Press, Bloomington, IN, 2008; pp. 159-165.
5. Dumont, M, Tafforeau, P, Bertin, T, Bhullar, BA, Field, D, Schulp, A, Strilisky, B, Thivichonprince, B, Viriot, L, & Louchart, A. Synchrotron imaging of dentition provides insights into the biology of *Hesperornis* and *Ichthyornis*, the “last” toothed birds. BMC Evolutionary Biology, 2016; 16: 178.
6. Hendrickx, C, Mateus O, Araújo R. A proposed terminology of theropod teeth (Dinosauria, Saurischia). Journal of Vertebrate Paleontology, 2015; e982797.
7. Howgate ME. The teeth of *Archaeopteryx* and a reinterpretation of the Eichstätt specimen. Zoological Journal of the Linnean Society. 1984; 82: 159–175.
8. Gao K, Brinkman DB. Choristoderes from the Park and in Its Vicinity. In Dinosaur Provincial Park, a spectacular ancient ecosystem revealed. Currie PJ, Koppelhus EB (editors). Indiana University Press, Bloomington, IN, 2005. pp. 221-234.
9. Gao K, Fox RC. New choristoderes (Reptilia: Diapsida) from the Upper Cretaceous and Paleocene, Alberta and Saskatchewan, Canada, and phylogenetic relationships of the Choristodera. Zoological Journal of the Linnean Society, 1998; 124: 303–353.
10. Matsumoto R, Evans SE. The palatal dentition of tetrapods and its functional significance. Journal of Anatomy, 2016; 228: 414-429.
11. **Larson, DW**. Diversity and variation of theropod dinosaur teeth from the uppermost Santonian Milk River Formation (Upper Cretaceous), Alberta: a quantitative method supporting identification of the oldest dinosaur tooth assemblage in Canada. Canadian Journal of Earth Sciences, 2008; 45: 1455-1468.
12. Larson, DW, Currie, PJ. Multivariate analyses of small theropod dinosaur teeth and implications for paleoecological turnover through time. PLoS One, 2013; 8: e54329.

Longrich NR. Small theropod teeth from the Lance Formation of Wyoming, USA. In: Sankey JT, Baszio, S, editors. Vertebrate Microfossil Assemblages: Their Role in Paleoecology and Paleobiogeography. Bloomington: Indiana University Press, 2008; pp. 135-158.

1. Louchart A, Pouech J. A tooth of Archaeopterygidae (Aves) from the Lower Cretaceous of France extends the spatial and temporal occurrence of the earliest birds. Cretaceous Research, 2017; 73: 30-36.
2. Mayr G, Burkhard P, Hartman S, Stefan Peters, D. The tenth skeletal specimen of Archaeopteryx. Zool J Linn Soc, 2007; 149 (1): 97-116.
3. Sankey, JT, Brinkman, DB, Guenther, M, Currie, PJ. Small theropod and bird teeth from Late Cretaceous (Late Campanian) Judith River group. Alberta Journal of Paleontology, 2002; 76: 751-63.
4. Smith, JB, Vann, DR, Dodson, P. Dental morphology and variation in theropod dinosaurs: Implications for the taxonomic identification of isolated teeth. Anat. Rec., 2005; 285A: 699-736.
5. Wang X, Zhao B, Shen C, Liu S, Gao C, Cheng X, Zhang F. New material of *Longipteryx* (Aves: Enantiornithes) from the Lower Cretaceous Yixian Formation of China with the first recognized avian tooth crenulations. Zootaxa, 2015; 3941: 565–578.
